# Histopathology-validated gross tumor volume delineations of intraprostatic lesions using PSMA-positron emission tomography/multiparametric magnetic resonance imaging

**DOI:** 10.1016/j.phro.2024.100633

**Published:** 2024-08-22

**Authors:** Josefine Grefve, Karin Söderkvist, Adalsteinn Gunnlaugsson, Kristina Sandgren, Joakim Jonsson, Angsana Keeratijarut Lindberg, Erik Nilsson, Jan Axelsson, Anders Bergh, Björn Zackrisson, Mathieu Moreau, Camilla Thellenberg Karlsson, Lars.E. Olsson, Anders Widmark, Katrine Riklund, Lennart Blomqvist, Vibeke Berg Loegager, Sara N. Strandberg, Tufve Nyholm

**Affiliations:** a Department of Diagnostics and Intervention, Radiation Physics, Umea University, Umea, Sweden; b Department of Diagnostics and Intervention, Oncology, Umea University, Umea, Sweden; c Department of Hematology, Oncology and Radiation Physics, Skane University Hospital, Lund University, Lund, Sweden; d Department of Diagnostics and Intervention, Diagnostic Radiology, Umea University, Umea, Sweden; e Department of Medical Biosciences, Pathology, Umea University, Umea, Sweden; f Department of Translational Medicine, Medical Radiation Physics, Lund University, Malmo, Sweden; g Department of Radiology, Copenhagen University Hospital in Herlev, Herlev, Denmark

## Abstract

**Background and purpose:**

Dose escalation in external radiotherapy of prostate cancer shows promising results in terms of biochemical disease-free survival. Boost volume delineation guidelines are sparse which may cause high interobserver variability. The aim of this research was to characterize gross tumor volume (GTV) delineations based on multiparametric magnetic resonance imaging (mpMRI) and prostate specific membrane antigen-positron emission tomography (PSMA-PET) in relation to histopathology-validated Gleason grade 4 and 5 regions.

**Material and methods:**

The study participants were examined with [^68^Ga]PSMA-PET/mpMRI prior to radical prostatectomy. Four radiation oncologists delineated GTVs in 15 study participants, on four different image types; T2-weighted (T2w), diffusion weighted imaging (DWI), dynamic contrast enhanced (DCE) and PSMA-PET scans separately. The simultaneous truth and performance level estimation (STAPLE) algorithm was used to generate combined GTVs. GTVs were subsequently compared to histopathology. We analysed how Dice similarity coefficient (DSC) and lesion coverage are affected by using single versus multiple image types as well as by adding a clinical target volume (CTV) margin.

**Results:**

Median DSC (STAPLE) for different GTVs varied between 0.33 and 0.52. GTV_PSMA-PET/mpMRI_ generated the highest median lesion coverage at 0.66. Combining different image types achieved similar lesion coverage as adding a CTV margin to contours from a single image type, while reducing non-malignant tissue inclusion within the target volume.

**Conclusion:**

The combined use of mpMRI or PSMA-PET/mpMRI shows promise, achieving higher DSC and lesion coverage while minimizing non-malignant tissue inclusion, in comparison to the use of a single image type with an added CTV margin.

## Highlights

• Volumetric assessments have been performed, comparing PSMA-positron emission tomography and magnetic resonance-based gross tumor volumes for intraprostatic lesions and histopathology.
• Combining multiparametric resonance imaging and PSMA-positron emission tomography enhances Dice similarity coefficient compared to other gross tumor volumes based on single modalities with added margins.
• Combining modalities is preferred for accurate intraprostatic lesion depiction.

## 1 Introduction

In radiotherapy of prostate cancer, the probability of biochemical disease-free survival increases with an increasing radiation dose, i.e. dose escalation. In patients with high risk of treatment failure, dose escalation is suggested to improve outcome [1], [2]. Increasing the dose to the entire prostate is associated with increased side effects [3], [4]. The implementation of a focal boost to the dominant intraprostatic lesion can offer a solution to dose escalation without excessive dose to nearby organs at risk. Focal boosting has been studied in the FLAME trial [5], a randomized controlled study investigating an integrated focal boost of 95 Gy in 35 fractions, while the rest of the prostate receives a dose of 77 Gy. The FLAME trial has demonstrated favorable results for patients receiving boost in terms of regional and distant metastasis free survival [6] and biochemical disease-free survival, with no excess impact on toxicity or quality of life.

At present the definition of the boost volume, commonly referred to as the gross tumor volume (GTV), primarily relies on medical imaging modalities such as magnetic resonance imaging (MRI) or positron emission tomography (PET) [7], [8], [9], [10]. Delineation of the boost volume based on multiple MRI sequences, termed multiparametric MRI (mpMRI), is the most common approach and includes T2-weighted (T2w), diffusion weighted imaging (DWI) and dynamic contrast enhanced (DCE) [8]. The Prostate Imaging Reporting and Data System (PI-RADS) is a guideline for image acquisition, detection, staging and diagnostic reporting of clinically relevant prostate cancer based on mpMRI [11]. With its wide implementation, PI-RADS has become a cornerstone in clinical diagnostics and management of prostate cancer. The PI-RADS recommendations for detection of intraprostatic regions are often referenced also in radiotherapy protocols for the delineation of GTV, even though PI-RADS is focused on detection rather than assessment of the extent of the disease. There are reports that mpMRI underestimates the extent of the tumor [7], [12], [13], [14], [15]. Prostate specific membrane antigen (PSMA)-PET can also be used to guide the delineation of intraprostatic lesions [8], [9]. PSMA-PET is believed to provide further complementary information to mpMRI [10], suggesting that a combination of PSMA-PET and mpMRI would more precisely delineate boost volumes.

There is a high subjectivity inherent in manual GTV delineations leading to interobserver variability [16], [17]. A retrospective analysis performed by the FLAME trial group demonstrated substantial variation in the GTV between institutes [18]. Detailed guidelines of regions to include or exclude in different imaging sequences are sparse which contributes to high interobserver variability.

One possible explanation for the lack of guidelines is the complex process to accurately verify the image findings. Numerous pitfalls warrant consideration, such as post biopsy hemorrhages, prostatitis and fibromuscular bands [19]. A significant challenge arises from the fact that the true lesion distribution is typically not known, thereby introducing difficulties in the image evaluation. However, this study uses whole mount histopathology slices with digitally delineated lesions, which provide a reliable ground truth, ensuring accurate assessment and validation of delineated targets.

The aim of this research was to characterize GTV delineations based on the following image types; T2w, DWI, DCE, and PSMA-PET in relation to regions with histopathology-validated Gleason grade 4 and 5. We also compared using single versus multiple image types as basis for the GTV delineation, and the addition of an intraprostatic clinical target volume (CTV) margin.

## 2 Material and methods

In this study, volumetric comparisons were conducted between GTV delineations on different image types and our reference standard, whole-mount histopathology with detailed lesion delineations of Gleason grade 4 and 5.

### 2.1 Study participants

In the present analysis 18 study participants were included. The study participants had high-risk prostate cancer and were planned for robotic assisted radical prostatectomy at Umea University Hospital. Approvals were obtained from the Regional Ethics board (DNR: 2016–220-31 M) and the Swedish medical products agency (EudraCT number: 2015–005046-55). Inclusion criteria were: ≥ 2 months since last biopsy of the prostate, age > 18 years, written informed consent, radiologically detected intraprostatic lesion [20], on either mpMRI or PSMA-PET (or both) and at least one histopathology slice within the dominant intraprostatic lesion with Gleason score ≥ 4 + 4. Consequently, slices with Gleason score 4 + 3 and 3 + 4 would also be included if they belonged to the same lesion. Exclusion criteria were contraindication to MRI or PET such as non-MR-safe implants or claustrophobia, World Health Organization performance status > 1, treatment with neoadjuvant/concomitant anti-testosterone treatment, TUR-P within 6 months and creatine clearance < 30 ml/min. All study participants were enrolled between December 2016 and December 2019. The study participants are a subset of the 55 study participants included in a previous publication by Sandgren et al. [20].

### 2.2 Data acquisition

Prior to radical prostatectomy, the study participants were examined with [^68^Ga]PSMA-PET/mpMRI (SIGNA PET/MR 3 T, GE Healthcare, Chicago, IL, USA). The mpMRI included the following sequences as specified in the PI-RADS v2 guideline [21]; T2w in three planes (axial, coronal and sagittal), T1w (axial), DCE, and DWI with b-values; b_0_ (0 s/mm^2^), b_200_ (200 s/mm^2^) and b_1000_ (1000 s/mm^2^). The DCE was acquired with a fast spoiled gradient-echo (FSPGR) sequence during 8 min with a frame acquisition time of 9.6 s, and 15 ml of Dotarem (Guerbet, Villepinte, France) was used as contrast agent. The image processing protocol used to calculate the K-trans and ADC parameter maps is described in Nilsson et al. [22]. For the PSMA-PET, 2 MBq/kg of [^68^Ga]PSMA-11 was intravenously injected 60 min prior to the examination. A static PSMA-PET was acquired simultaneously with the MRI for 41 min and reconstructed with SharpIR (GE Healthcare) an iterative resolution-enhanced ordered subset expectation maximization (OSEM)-based algorithm. The slice thickness was 2.5 mm for the axial T2w, 3 mm for the sagittal and coronal T2w, 5 mm for the DWI and DCE, and 2.78 mm for the PSMA-PET. Full study protocol has previously been presented by Nilsson et al. [22].

Directly following excision, the prostate specimen was placed in a 3D-printed custom-made mold. The mold was used to prevent specimen deformation after excision and constructed with built-in slits aligned with the MR imaging planes to give same orientation in the histopathology sectioning. Prior to formalin fixation, the prostate specimen was scanned ex-vivo inside its mold with a T2w sequence using the same MRI scanner as for the in-vivo imaging. The specimen was sliced at least 24 h after formalin fixation into 5 mm thick slices, using the built-in slits of the prostate mold as guidance. The method is described in detail by Sandgren et al. [23].

Paraffin embedding and micro-toming into 5 µm thick slices was followed by glass mounting and staining with Hematoxylin and Eosin (H&E). A pathologist (>30 y of experience) digitally delineated tumor foci using NDP.view2 (Hamamatsu Photonic K.K, Hamamatsu city, Japan) and graded the lesions according to the Gleason grading system [24].

### 2.3 Delineation protocol

To ensure consistent and high-quality delineation, a draft guideline for delineating intraprostatic lesions was created based on the PI-RADS v2.1 guideline for the MR image types [11]. The draft was evaluated during a workshop by four experienced radiation oncologists (>10 y of experience). Three patients (with a highest recorded Gleason score of 4 + 4) were used as a practice set during this session and the remaining 15 were used in the analysis.

In the final delineation guideline, tumorous regions were defined as lenticular or non-circumscribed, homogeneous, moderately hypointense foci on T2w, focal markedly hypointense on ADC and markedly hyperintense on high b-value DWI (b_1000_), regions with early contrast enhancement on DCE and/or distinct regions with elevated K-trans or as focal markedly increased uptake in PSMA-PET (window level SUV 0–10).

### 2.4 GTV delineations

The GTVs in the remaining cases were delineated in Oncentra (Elekta AB, Stockholm, Sweden) and Eclipse (Varian Medical System, Palo Alto, CA), under the assumption that these volumes were going to be used as intraprostatic boost volumes. The radiological report, including coordinates based on the PI-RADS v2.1 sector map [11], zone, PI-RADS score, PROMISE score, and PSMA-RADS score were provided during the delineations. Each observer delineated four GTVs on each patient, based on T2w, DWI, DCE and PSMA-PET images separately. The observers were allowed to review all the material prior to delineating GTVs in each image type.

### 2.5 Image registrations

The histopathology slices were registered to the in-vivo images using the procedure described in [23], yielding a median in-plane registration error of 1.7 mm. After registration, the histopathology delineations that overlapped in the z-direction, in consecutive slices, were considered as the same lesion.

To evaluate the impact of the registration error, an additional experiment was performed with the goal of optimizing overlap measures while adhering to the known registration error limit of 1.7 mm. This optimization involved a translation registration of the in-vivo GTVs to the histopathology delineations, employing the kappa statistic metric to maximize the Dice similarity coefficient (DSC) while imposing a penalty term on the translation distance. The weighting of this penalty term was adjusted to ensure that the average translation distance across the entire patient population remained at 1.7 mm after this step. The optimal weight was computed using a combined GTV based on all MRI sequences and physicians, created by the simultaneous truth and performance level estimation (STAPLE) algorithm [25]. STAPLE is a well-established method that provides a probabilistic estimate of the true segmentation from a collection of segmentations of the same structure, and it is easily accessible through resources such as the open-source library Insight Segmentation and Registration Toolkit (ITK).

### 2.6 Volumetric comparisons

The GTVs from the four observers were combined for each image type using the STAPLE algorithm in Hero (Hero imaging AB, Umeå, Sweden). This resulted in four different GTVs for each study participant, all of which were generated by thresholding the ground truth estimation output from the STAPLE filter at 0.95, denoted as GTV_T2w_, GTV_DWI_, GTV_DCE_, and GTV_PSMA-PET_.

**Fig. 1:**
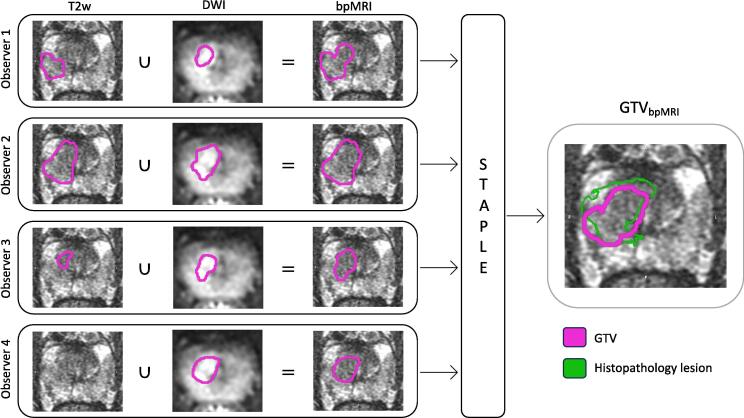
GTV_bpMRI_ were created in two steps. First the union of individual observer delineations were taken (T2w and DWI), and secondly the STAPLE algorithm was used to combine the GTV unions from the four observers.

The GTVs for combinations of multiple image types (bi-parametric MRI (bpMRI), mpMRI, and PSMA-PET/mpMRI) were created in two steps − first the individual observer delineations were combined into a union, and secondly the STAPLE algorithm was used to combine the GTV unions from the four observers. Fig. 1 illustrates the creation of GTV_bpMRI_, including T2w and DWI. The same procedure was applied for GTV_mpMRI_ (T2w, DWI, and DCE) and for GTV_PSMA-PET/mpMRI_ (all four image types). If any of the GTVs extended beyond the histopathology border, they were cropped to include solely the regions where ground truth histopathology was present. This adjustment was made to exclude the influence of extraprostatic regions in our analysis.

DSC and lesion coverage (voxel-wise sensitivity calculated as the quotient between the intersection of the GTV with the histopathology lesion and the histopathology lesion) measures between resulting GTVs and histopathology lesions were calculated for both single and multiple image types. Additionally, we studied the benefit of adding isotropic margins (CTVs) to GTVs from single and multiple image types. To assess the interobserver variability, the overlap resulting from the individual observers' delineations was also calculated. The combined GTVs for each individual observer were created by taking the union of the delineations made in the different image types.

## 3 Results

**Fig. 2:**
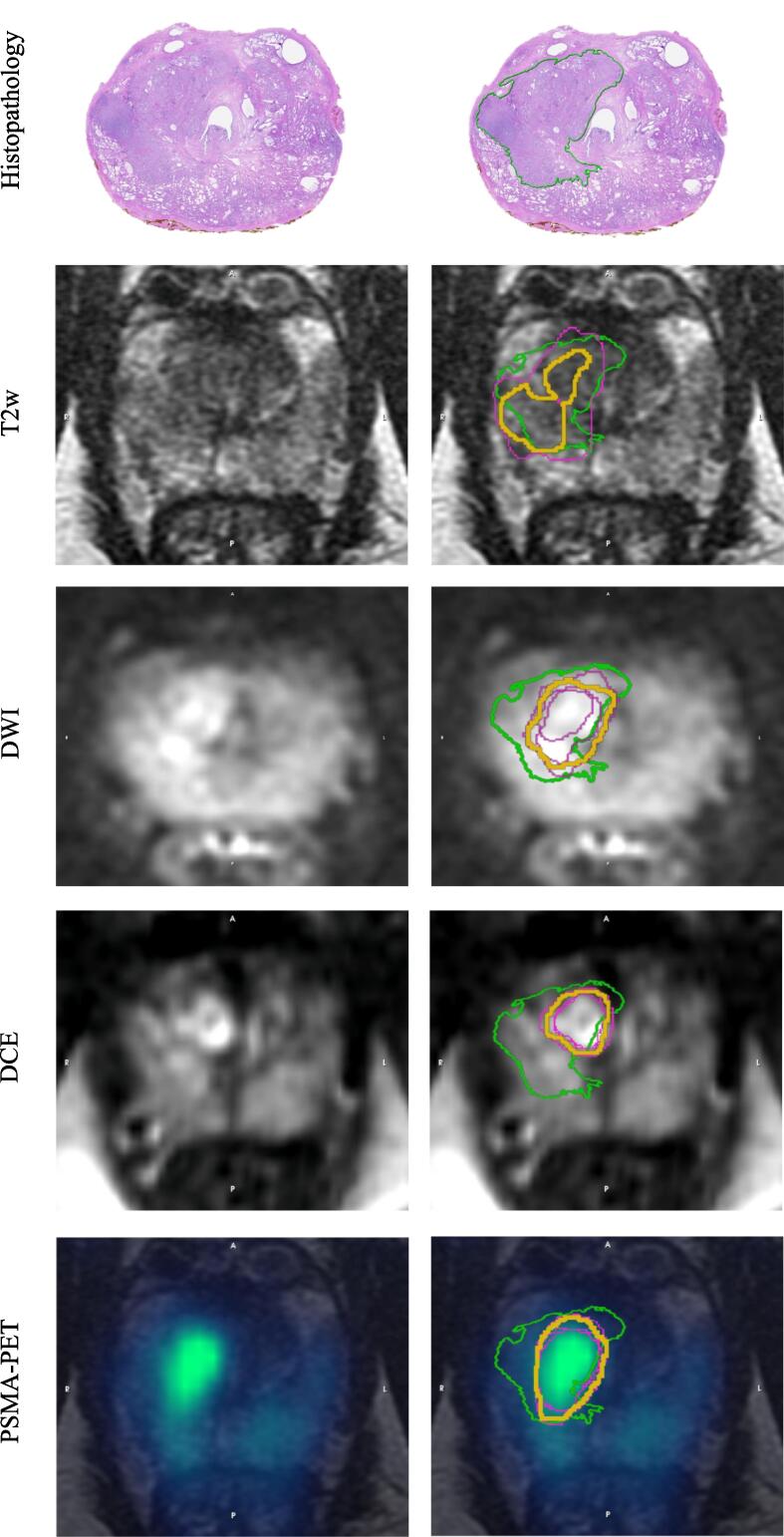
Histopathology lesion (green line), the 4 observers individual GTV delineations (purple lines) and the combined GTV from the STAPLE algorithm (yellow line). (For interpretation of the references to colour in this figure legend, the reader is referred to the web version of this article.)

Patient characteristics are presented in Table 1. The median lesion volume of Gleason grade 4 and 5 regions was 1.64 ml (range 0.22–11.22 ml). Fig. 2 presents a representative histopathology slice, including the observers individual GTVs and combined GTV for the different image types.

**Table 1:** Characteristics of the 15 included study participants. N=number of patients, RP=radical prostatectomy. The Gleason score corresponds to the highest recorded Gleason score in the dominant intraprostatic lesion.

**Characteristics**	**Median (min, max)**
Age [years]	65 (54, 76)
Weight [kg]	85 (69, 100)
Lesion volume total [ml]	2.21 (0.52, 11.69)
Lesion volume comprised of Gleason grade 4 & 5 [ml]	1.64 (0.22, 11.22)
PSA [ng/ml]	6.5 (3.9, 13.3)
Days between imaging and surgery	23 (2, 59)
Injected activity PSMA [MBq]	166 (138, 181)
**Post RP Gleason score**	**N (%)**
4 + 4	12 (80 %)
4 + 5	1 (6.7 %)
5 + 4	2 (13.3 %)
5 + 5	0 (0 %)
**pT status**	
T2a	3 (20 %)
T3b	12 (80 %)

**Fig. 3:**
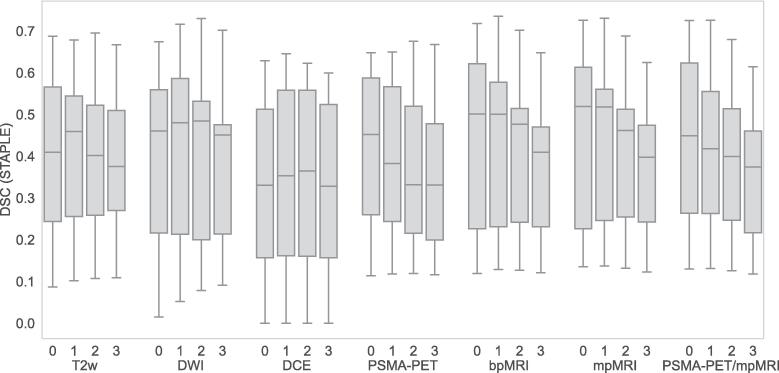
DSC distribution GTV (STAPLE) with CTV margins 0, 1, 2 and 3 mm (Gleason grade regions 4 and 5). The boxplot represents the minimum, first quartile, median, third quartile and maximum DSC value.

**Fig. 4:**
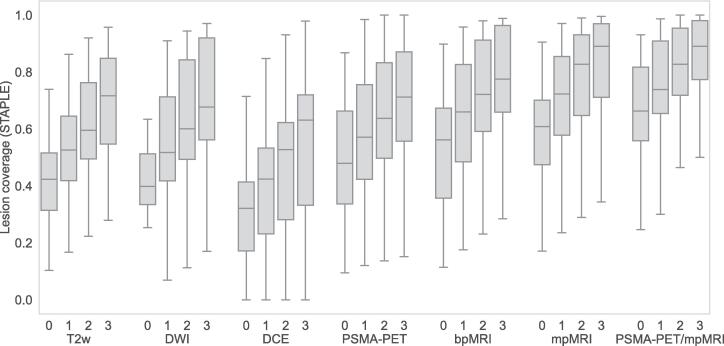
Lesion coverage distribution GTV (STAPLE) with CTV margins 0, 1, 2 and 3 mm (Gleason grade regions 4 and 5). The boxplot represents the minimum, first quartile, median, third quartile and maximum lesion coverage value.

Table 2 shows DSC for the different GTVs and the corresponding lesion coverage. The highest median DSC was obtained by combining multiple image types. The median DSC (STAPLE) without CTV margin was 0.41 (GTV_T2w_), 0.46 (GTV_DWI_), 0.33 (GTV_DCE_), 0.45 (GTV_PSMA-PET_), 0.50 (GTV_bpMRI_), 0.52 (GTV_mpMRI_) and 0.45 (GTV_PSMA-PET/mpMRI_). The highest median lesion coverage (STAPLE) without CTV margin was 0.66 found in GTV_PSMA-PET/mpMRI_. The mean of the individual observers’ median DSC without CTV margin was 0.36 (GTV_T2w_), 0.35 (GTV_DWI_), 0.26 (GTV_DCE_), 0.42 (GTV_PSMA-PET_), 0.42 (GTV_bpMRI_), 0.46 (GTV_mpMRI_) and 0.46 (GTV_PSMA-PET/mpMRI_) and the highest lesion coverage value was 0.61 (GTV_PSMA-PET/mpMRI_). Fig. 3, Fig. 4 depicts the DSC and the lesion coverage distribution (GTV STAPLE), respectively, and shows the high variability between the patients.

**Table 2:** DSC and lesion coverage comparing GTV with histopathology lesion with CTV margins 0, 1, 2 and 3 mm (Gleason grade regions 4 and 5). For GTV STAPLE (S), the median, maximum and minimum values of the 15 study participants are shown. For GTV Individual (I), the mean value calculated from the 4 observers' median values, as well as the maximum and minimum median value, are shown. Therefore, the ranges for the STAPLE and individual GTVs are not comparable.

**DSC Median (min, max)**				
	0 mm	1 mm	2 mm	3 mm
T2w (S)	0.41 (0.09, 0.69)	0.46 (0.10, 0.68)	0.40 (0.11, 0.70)	0.38 (0.11, 0.67)
T2w (I)	0.36 (0.26, 0.46)	0.39 (0.26, 0.52)	0.37 (0.28, 0.48)	0.35 (0.29, 0.43)
DWI (S)	0.46 (0.02, 0.67)	0.48 (0.05, 0.72)	0.48 (0.08, 0.73)	0.45 (0.09, 0.70)
DWI (I)	0.35 (0.23, 0.48)	0.40 (0.33, 0.49)	0.42 (0.37, 0.48)	0.41 (0.39, 0.43)
DCE (S)	0.33 (0.00, 0.63)	0.35 (0.00, 0.65)	0.36 (0.00, 0.62)	0.33 (0.00, 0.60)
DCE (I)	0.26 (0.18, 0.46)	0.28 (0.20, 0.45)	0.28 (0.23, 0.39)	0.29 (0.24, 0.37)
PSMA-PET (S)	0.45 (0.11, 0.65)	0.38 (0.12, 0.65)	0.33 (0.12, 0.68)	0.33 (0.12, 0.67)
PSMA-PET (I)	0.42 (0.36, 0.49)	0.39 (0.38, 0.42)	0.35 (0.32, 0.40)	0.32 (0.31, 0.34)
bpMRI (S)	0.50 (0.12, 0.72)	0.50 (0.13, 0.74)	0.48 (0.13, 0.70)	0.41 (0.12, 0.65)
bpMRI (I)	0.42 (0.25, 0.55)	0.42 (0.28, 0.51)	0.41 (0.32, 0.46)	0.36 (0.35, 0.37)
mpMRI (S)	0.52 (0.13, 0,73)	0.52 (0.14, 0.73)	0.46 (0.13, 0.69)	0.40 (0.12, 0.62)
mpMRI (I)	0.46 (0.37, 0.55)	0.44 (0.37, 0.50)	0.40 (0.36, 0.44)	0.39 (0.33, 0.42)
PSMA-PET/mpMRI (S)	0.45 (0.13, 0.73)	0.42 (0.13, 0.73)	0.40 (0.13, 0.68)	0.37 (0.12, 0.61)
PSMA-PET/mpMRI (I)	0.46 (0.42, 0.49)	0.42 (0.40, 0.46)	0.40 (0.38, 0.43)	0.36 (0.35, 0.38)

**Lesion coverage Median (min, max)**				
	0 mm	1 mm	2 mm	3 mm

T2w (S)	0.42 (0.10, 0.74)	0.53 (0.17, 0.86)	0.60 (0.22, 0.92)	0.72 (0.28, 0.96)
T2w (I)	0.37 (0.20, 0.57)	0.45 (0.25, 0.67)	0.52 (0.31, 0.76)	0.57 (0.34, 0.83)
DWI (S)	0.40 (0.02, 0.83)	0.52 (0.07, 0.91)	0.60 (0.11, 0.94)	0.68 (0.17, 0.97)
DWI (I)	0.34 (0.22, 0.48)	0.45 (0.31, 0.59)	0.55 (0.40, 0.73)	0.62 (0.49, 0.77)
DCE (S)	0.32 (0.00, 0.71)	0.42 (0.00, 0.85)	0.53 (0.00, 0.93)	0.63 (0.00, 0.98)
DCE (I)	0.26 (0.20, 0.33)	0.35 (0.26, 0.42)	0.45 (0.34, 0.51)	0.50 (0.43, 0.53)
PSMA-PET (S)	0.48 (0.09, 0.87)	0.57 (0.12, 0.99)	0.64 (0.14, 1.00)	0.71 (0.15, 1.00)
PSMA-PET (I)	0.44 (0.24, 0.58)	0.54 (0.35, 0.65)	0.61 (0.45, 0.72)	0.68 (0.54, 0.79)
bpMRI (S)	0.56 (0.11, 0.90)	0.66 (0.18, 0.96)	0.72 (0.23, 0.98)	0.78 (0.28, 0.99)
bpMRI (I)	0.48 (0.37, 0.66)	0.58 (0.46, 0.76)	0.67 (0.56, 0.86)	0.74 (0.65, 0.90)
mpMRI (S)	0.61 (0.17, 0.91)	0.72 (0.24, 0.97)	0.83 (0.29, 0.99)	0.89 (0.34, 1.00)
mpMRI (I)	0.53 (0.39, 0.67)	0.66 (0.52, 0.76)	0.77 (0.62, 0.86)	0.84 (0.73, 0.91)
PSMA-PET/mpMRI (S)	0.66 (0.25, 0.93)	0.74 (0.30, 0.99)	0.83 (0.35, 1.00)	0.89 (0.40, 1.00)
PSMA-PET/mpMRI (I)	0.61 (0.48, 0.77)	0.71 (0.60, 0.84)	0.81 (0.70, 0.88)	0.88 (0.80, 0.94)

Adding CTV margins to individual image types resulted in comparable lesion coverage values to those obtained by the combined image types without margins. However, this approach also led to a higher proportion of non-malignant tissue being included, resulting in lower DSC.

To assess the impact of the known registration uncertainty on the results, an additional translation transform was applied which displaced the GTVs (STAPLE) slightly. The median DSC increased between 0.04 and 0.07 and the median lesion coverage increased between 0.02 and 0.16 for the different image types. Consistent with the previous results, GTV_bpMRI_, GTV_mpMRI_ and GTV_PSMA-PET/mpMRI_ generated higher median DSC than GTVs based on individual image types. GTV_mpMRI_ without CTV margin generated the highest median DSC. DSC and lesion coverage values for the additional translation are presented in Supplement 1.

## 4 Discussion

In this study we characterized GTV delineations based on T2w, DWI, DCE and PSMA-PET images in relation to Gleason grade 4 and 5 regions delineated on whole-mount histopathology. Our analysis indicates that using a combination of imaging types (e.g., T2w, DWI, and DCE; or PSMA-PET and mpMRI) achieves comparable lesion coverage to that of adding a CTV margin to contours made using a single imaging type (e.g., T2w alone). Furthermore, combining different image types resulted in a lower proportion of non-malignant prostate tissue in the target. The highest median lesion coverage without CTV margin was found from PSMA-PET/mpMRI. The benefit of using a combined PSMA-PET/mpMRI as basis for GTV boost volume delineations is in line with other publications [8], [10], [26].

In our study, the median DSC varied between 0.33 and 0.52 for the different GTVs (STAPLE) where the highest value was found for GTV_mpMRI_. In terms of lesion coverage, the median values varied between 0.32 and 0.66. The mean of the individual observers’ median DSC varied between 0.26 and 0.46 and the lesion coverage varied between 0.26 and 0.61. Both the DSC and the lesion coverage values are slightly lower for the individual GTVs compared to the STAPLE GTVs. STAPLE provides an approximation of the achievable overlap, while the range of individual GTVs reflects the interobserver variability. Consistent for both the STAPLE GTVs and the individual GTVs was that GTV_PSMA-PET/mpMRI_ generated higher median lesion coverage as compared to GTV_mpMRI_ alone. Additionally, the lowest interobserver variability was found for GTV_PSMA-PET/mpMRI_ using the individual GTVs. Adding CTV margins to individual image types resulted in lesion coverage values comparable to the GTVs created from multiple image types. However, this approach led to the inclusion of more non-malignant tissue within the GTV, consequently resulting in a decrease in DSC. Substantial variability was evident among patients, with the DSC for the GTV_PSMA-PET/mpMRI_ ranging from 0.13 to 0.73. The histopathology lesion volume of Gleason grade 4 and 5 regions also varied greatly, with a median of 1.64 ml (range 0.22–11.22 ml).

Betterman et al. [8] reported higher sensitivity values as compared to our lesion coverage values. However, it is important to highlight that Betterman et al. performed a quadrant-based analysis, whereas our study performed a voxel-wise comparison. Also, their patient cohort had larger lesion volumes (median 10.4 ml), that together with higher PSA values (median 17.4 ng/ml), could indicate higher risk characteristics of the population as compared to ours, which could make lesions easier to define.

Given the inherent in-plane registration error of 1.7 mm we performed an examination of the potential impact of this on our results by introducing an additional translation transform to the previously registered histopathology lesions. The median DSC increased between 0.04 and 0.07, however, this did not impact the conclusion of the study, i.e. GTVs from bpMRI, mpMRI and PSMA-PET/mpMRI resulted in higher DSC.

A window level of SUV 0–10 was used for the PSMA-PET based delineations. Other studies [27] used a window level of 0–5, but this setting did not yield satisfactory results on our data. One possible explanation for the need of different window settings may be the use of different reconstruction parameters. Resolution modeling (SharpIR) was used for our data set which might lead to higher SUV-max values, leading to a need for wider window settings.

One limitation of this study is the relatively small number of patients included. Although the original dataset consisted of 55 patients, including both intermediate and high-risk prostate cancer cases, we narrowed our focus to solely incorporate patients with a Gleason score of ≥ 4 + 4, as those with higher Gleason scores are likely to benefit the most from a boost [28]. Furthermore, the histopathology delineations were performed by a single pathologist with extensive experience (>30 years). While this ensures consistent delineations between cases, we have not assessed or accounted for any inter- or intra-observer variations in the histology delineations.

Additionally, this study is constrained by the timing of mpMRI data acquisition, which occurred in December 2016, predating the release of the PI-RADS v2.1 guideline. This updated guideline currently recommends a slice thickness of 3 mm for both diffusion-weighted and contrast-enhanced sequences. Our dataset utilized a 5 mm slice thickness to facilitate correlation between in-vivo imaging and histopathology sections, thus deviating from the current guideline recommendations. In future studies a 3 mm slice thickness can be employed. We found that even the most comprehensive coverage, using all the images with an added 3 mm CTV margin, did not fully encompass the whole tumor. While the FLAME study [5] achieved positive outcomes without additional CTV margins, i.e. their GTVs are likely even smaller, the significance of fully encompassing the tumor within the boost volume remains uncertain. Our results are based on the delineations made by four oncologists from two different sites and other oncologist may interpret the data differently. However, by prefacing the delineations with constructing a consensus-based delineation guideline, we aimed to mitigate the inter-observer variability to the greatest possible extent.

In conclusion, our study suggests that using multiple image types is beneficial for accurate GTV delineation in prostate cancer. The combined use of mpMRI or PSMA-PET/mpMRI shows promise, achieving higher DSC and lesion coverage while minimizing non-malignant tissue inclusion, in comparison to the use of a single image type with an added CTV margin.

## Declaration of Competing Interest

This work was funded by Swedish Cancer Society, Cancer research foundation of Northen Sweden, Prostatacancerförbundet and Västerbotten County. Furthermore, Joakim Jonsson and Tufve Nyholm are part-owner in Hero Imaging AB which produces the software HERO that were used in the analysis.
