# Supplementary material for: Histopathology-validated gross tumor volume delineations of intraprostatic lesions using PSMA-positron emission tomography/multiparametric magnetic resonance imaging
Source: Phys Imaging Radiat Oncol. 2024 Aug 22;31:100633. doi: 10.1016/j.phro.2024.100633 (PMC11402543; doi:10.1016/j.phro.2024.100633)
Supplement: Supplementary Data 1 [file mmc1.docx]

Supplementary 1: Table 1: DSC comparing GTV (STAPLE) with histopathology lesion with CTV margins 0, 1, 2 and 3 mm (Gleason grade regions 4 and 5).

| **Resulting DSC Median (min, max) after additional translation transform** | | | | |
| --- | --- | --- | --- | --- |
|  | 0 mm | 1 mm | 2 mm | 3 mm |
| T2w | 0.48 (0.13, 0.71) | 0.48 (0.18, 0.71) | 0.45 (0.19, 0.70) | 0.42 (0.16, 0.66) |
| DWI | 0.50 (0.08, 0.75) | 0.52 (0.11, 0.77) | 0.52 (0.11, 0.76) | 0.46 (0.10, 0.71) |
| DCE | 0.38 (0.00, 0.63) | 0.43 (0.00, 0.75) | 0.40 (0.00, 0.72) | 0.36 (0.00, 0.65) |
| PSMA-PET | 0.51 (0.14, 0.70) | 0.42 (0.22, 0.71) | 0.42 (0.21, 0.72) | 0.42 (0.18, 0.69) |
| bpMRI | 0.54 (0.20, 0.80) | 0.54 (0.20, 0.79) | 0.51 (0.16, 0.73) | 0.44 (0.13, 0.65) |
| mpMRI | 0.56 (0.21, 0,81) | 0,55 (0.19, 0.77) | 0.48 (0.15, 0.71) | 0.42 (0.13, 0.63) |
| PSMA-PET/mpMRI | 0.52 (0.23, 0.80) | 0.47 (0.17, 0.76) | 0.44 (0.14, 0.70) | 0.41 (0.12, 0.62) |

Table 2: Lesion coverage between GTV (STAPLE) and histopathology lesion with CTV margins 0, 1, 2 and 3 mm (Gleason grade regions 4 and 5).

| **Resulting Lesion coverage Median (min, max) after additional translation transform** | | | | |
| --- | --- | --- | --- | --- |
|  | 0 mm | 1 mm | 2 mm | 3 mm |
| T2w | 0.53 (0.19, 0.74) | 0.67 (0.27, 0.88) | 0.76 (0.33, 0.95) | 0.81 (0.39, 0.98) |
| DWI | 0.42 (0.09, 0.87) | 0.62 (0.15, 0.94) | 0.68 (0.20, 0.96) | 0.74 (0.20, 1.00) |
| DCE | 0.41 (0.00, 0.74) | 0.53 (0.00, 0.90) | 0.62 (0.00, 0.96) | 0.68 (0.00, 0.99) |
| PSMA-PET | 0.55 (0.08, 0.96) | 0.68 (0.14, 1.00) | 0.76 (0.16, 1.00) | 0.81 (0.18, 1.00) |
| bpMRI | 0.65 (0.20, 0.93) | 0.78 (0.28, 0.98) | 0.85 (0.34, 0.99) | 0.91 (0.40, 1.00) |
| mpMRI | 0.72 (0.27, 0.94) | 0.89 (0.34, 0.98) | 0.94 (0.40, 0.99) | 0.97 (0.46, 1.00) |
| PSMA-PET/mpMRI | 0.83 (0.34, 0.96) | 0.91 (0.41, 1.00) | 0.95 (0.46, 1.00) | 0.97 (0.52, 1.00) |
