# Supplementary material for: Histopathology-validated gross tumor volume delineations of intraprostatic lesions using PSMA-positron emission tomography/multiparametric magnetic resonance imaging
Source: Phys Imaging Radiat Oncol. 2024 Aug 22;31:100633. doi: 10.1016/j.phro.2024.100633 (PMC11402543; doi:10.1016/j.phro.2024.100633)
Supplement: Supplementary Data 2 [file mmc2.docx]

Supplementary 2: Table 1 DSC comparing GTV with histopathology delineation with CTV margins 0, 1, 2 and 3 mm (Gleason grade regions 4 and 5).

| **DSC Median (min, max)** | | | | | |
| --- | --- | --- | --- | --- | --- |
|  | Observer | 0 mm | 1 mm | 2 mm | 3 mm |
| T2w | 1 | 0.40 (0.00, 0.66) | 0.43 (0.00, 0.63) | 0.41 (0.00, 0.60) | 0.39 (0.00, 0.57) |
|  | 2 | 0.31 (0.00, 0.70) | 0.33 (0.00, 0.70) | 0.33 (0.00, 0.68) | 0.31 (0.00, 0.62) |
|  | 3 | 0.46 (0.00, 0.67) | 0.52 (0.00, 0.66) | 0.48 (0.00, 0.61) | 0.43 (0.00, 0.63) |
|  | 4 | 0.26 (0.00, 0.71) | 0.26 (0.00, 0.66) | 0.28 (0.00, 0.60) | 0.29 (0.00, 0.53) |
| DWI | 1 | 0.37 (0.02, 0.63) | 0.41 (0.07, 0.64) | 0.43 (0.10, 0.67) | 0.43 (0.12, 0.65) |
|  | 2 | 0.23 (0.01, 0.66) | 0.33 (0.05, 0.71) | 0.40 (0.08, 0.70) | 0.40 (0.09, 0.66) |
|  | 3 | 0.48 (0.13, 0.69) | 0.49 (0.15, 0.69) | 0.48 (0.15, 0.71) | 0.41 (0.14, 0.71) |
|  | 4 | 0.32 (0.00, 0.69) | 0.35 (0.00, 0.66) | 0.37 (0.02, 0.70) | 0.39 (0.05, 0.71) |
| DCE | 1 | 0.46 (0.00, 0.57) | 0.45 (0.00, 0.59) | 0.39 (0.00, 0.61) | 0.37 (0.00, 0.60) |
|  | 2 | 0.20 (0.00, 0.62) | 0.24 (0.00, 0.64) | 0.27 (0.00, 0.62) | 0.29 (0.00, 0.59) |
|  | 3 | 0.22 (0.00, 0.57) | 0.23 (0.00, 0.64) | 0.24 (0.00, 0.63) | 0.24 (0.00, 0.59) |
|  | 4 | 0.18 (0.00, 0.58) | 0.20 (0.00, 0.60) | 0.23 (0.00, 0.62) | 0.24 (0.00, 0.60) |
| PSMA-PET | 1 | 0.49 (0.07, 0.63) | 0.42 (0.09, 0.66) | 0.36 (0.11, 0.69) | 0.31 (0.12, 0.66) |
|  | 2 | 0.36 (0.05, 0.66) | 0.38 (0.08, 0.65) | 0.40 (0.10, 0.63) | 0.34 (0.10, 0.66) |
|  | 3 | 0.44 (0.11, 0.64) | 0.38 (0.11, 0.61) | 0.32 (0.11, 0.65) | 0.32 (0.11, 0.66) |
|  | 4 | 0.39 (0.00, 0.64) | 0.38 (0.00, 0.65) | 0.33 (0.00, 0.68) | 0.32 (0.00, 0.68) |

Table 2 DSC comparing GTV with histopathology delineation with CTV margins 0, 1, 2 and 3 mm (Gleason grade regions 4 and 5).

| **DSC Median (min, max)** | | | | | |
| --- | --- | --- | --- | --- | --- |
|  | Observer | 0 mm | 1 mm | 2 mm | 3 mm |
| bpMRI | 1 | 0.44 (0.01, 0.65) | 0.45 (0.05, 0.70) | 0.45 (0.07, 0.68) | 0.42 (0.08, 0.63) |
|  | 2 | 0.25 (0.10, 0.74) | 0.28 (0.11, 0.72) | 0.32 (0.11, 0.67) | 0.33 (0.11, 0.61) |
|  | 3 | 0.55 (0.11, 0.69) | 0.51 (0.13, 0.69) | 0.46 (0.13, 0.70) | 0.41 (0.12, 0.67) |
|  | 4 | 0.43 (0.09, 0.71) | 0.42 (0.11, 0.68) | 0.41 (0.11, 0.71) | 0.39 (0.09, 0.71) |
| mpMRI | 1 | 0.48 (0.11, 0.68) | 0.46 (0.12, 0.71) | 0.41 (0.12, 0.68) | 0.38 (0.12, 0.62) |
|  | 2 | 0.37 (0.10, 0.75) | 0.37 (0.10, 0.73) | 0.36 (0.11, 0.67) | 0.34 (0.11, 0.60) |
|  | 3 | 0.55 (0.13, 0.69) | 0.50 (0.13, 0.69) | 0.44 (0.13, 0.68) | 0.41 (0.12, 0.64) |
|  | 4 | 0.43 (0.15, 0.71) | 0.42 (0.17, 0.70) | 0.40 (0.16, 0.70) | 0.39 (0.15, 0.66) |
| PSMA-PET/mpMRI | 1 | 0.49 (0.11, 0.69) | 0.42 (0.12, 0.70) | 0.38 (0.12, 0.66) | 0.35 (0.11, 0.60) |
|  | 2 | 0.42 (0.09, 0.75) | 0.40 (0.10, 0.72) | 0.40 (0.10, 0.67) | 0.36 (0.10, 0.60) |
|  | 3 | 0.48 (0.13, 0.69) | 0.46 (0.13, 0.70) | 0.43 (0.12, 0.68) | 0.36 (0.11, 0.64) |
|  | 4 | 0.44 (0.17, 0.70) | 0.42 (0.17, 0.70) | 0.40 (0.16, 0.70) | 0.38 (0.14, 0.65) |

Table 3 Lesion coverage between GTV and histopathology lesion with CTV margins 0, 1, 2 and 3 mm (Gleason grade regions 4 and 5).

| **Lesion coverage Median (min, max)** | | | | | |
| --- | --- | --- | --- | --- | --- |
|  | Observer | 0 mm | 1 mm | 2 mm | 3 mm |
| T2w | 1 | 0.36 (0.00, 0.70) | 0.43 (0.00, 0.82) | 0.49 (0.00, 0.89) | 0.54 (0.00, 0.93) |
|  | 2 | 0.36 (0.00, 0.66) | 0.45 (0.00, 0.77) | 0.52 (0.00, 0.85) | 0.57 (0.00, 0.94) |
|  | 3 | 0.57 (0.00, 0.88) | 0.67 (0.00, 0.95) | 0.76 (0.00, 0.98) | 0.83 (0.00, 1.00) |
|  | 4 | 0.20 (0.00, 0.76) | 0.25 (0.00, 0.85) | 0.31 (0.00, 0.90) | 0.34 (0.00, 0.92) |
| DWI | 1 | 0.35 (0.01, 0.80) | 0.47 (0.07, 0.89) | 0.57 (0.13, 0.96) | 0.65 (0.18, 0.99) |
|  | 2 | 0.22 (0.01, 0.74) | 0.31 (0.04, 0.86) | 0.40 (0.07, 0.93) | 0.49 (0.11, 0.97) |
|  | 3 | 0.48 (0.14, 0.81) | 0.59 (0.19, 0.91) | 0.73 (0.25, 0.94) | 0.77 (0.31, 0.98) |
|  | 4 | 0.31 (0.00, 0.81) | 0.42 (0.00, 0.91) | 0.48 (0.04, 0.94) | 0.56 (0.06, 0.97) |
| DCE | 1 | 0.33 (0.00, 0.85) | 0.41 (0.00, 0.91) | 0.51 (0.00, 0.96) | 0.53 (0.00, 0.99) |
|  | 2 | 0.20 (0.00, 0.73) | 0.26 (0.00, 0.85) | 0.34 (0.00, 0.93) | 0.43 (0.00, 0.98) |
|  | 3 | 0.32 (0.00, 0.56) | 0.42 (0.00, 0.69) | 0.51 (0.00, 0.80) | 0.53 (0.00, 0.88) |
|  | 4 | 0.20 (0.00, 0.75) | 0.32 (0.00, 0.82) | 0.42 (0.00, 0.88) | 0.53 (0.00, 0.92) |
| PSMA-PET | 1 | 0.51 (0.09, 0.94) | 0.59 (0.12, 1.00) | 0.66 (0.14, 1.00) | 0.72 (0.15, 1.00) |
|  | 2 | 0.24 (0.05, 0.77) | 0.35 (0.10, 0.87) | 0.45 (0.16, 0.94) | 0.54 (0.20, 0.99) |
|  | 3 | 0.58 (0.09, 0.87) | 0.65 (0.12, 0.99) | 0.72 (0.14, 1.00) | 0.79 (0.15, 1.00) |
|  | 4 | 0.42 (0.00, 0.83) | 0.55 (0.00, 0.92) | 0.63 (0.00, 0.98) | 0.68 (0.00, 1.00) |

Table 4 Lesion coverage between GTV and histopathology lesion with CTV margins 0, 1, 2 and 3 mm (Gleason grade regions 4 and 5).

| **Lesion coverage Median (min, max)** | | | | | |
| --- | --- | --- | --- | --- | --- |
|  | Observer | 0 mm | 1 mm | 2 mm | 3 mm |
| bpMRI | 1 | 0.50 (0.01, 0.88) | 0.63 (0.07, 0.94) | 0.70 (0.13, 0.98) | 0.76 (0.19, 0.99) |
|  | 2 | 0.37 (0.10, 0.78) | 0.46 (0.16, 0.89) | 0.56 (0.22, 0.95) | 0.66 (0.28, 0.99) |
|  | 3 | 0.66 (0.17, 0.88) | 0.76 (0.23, 0.95) | 0.86 (0.28, 0.98) | 0.90 (0.34, 1.00) |
|  | 4 | 0.37 (0.08, 0.88) | 0.46 (0.13, 0.95) | 0.57 (0.19, 0.97) | 0.65 (0.20, 0.99) |
| mpMRI | 1 | 0.54 (0.19, 0.93) | 0.66 (0.26, 0.97) | 0.76 (0.31, 0.99) | 0.85 (0.36, 1.00) |
|  | 2 | 0.51 (0.12, 0.82) | 0.70 (0.21, 0.92) | 0.81 (0.26, 0.97) | 0.88 (0.30, 1.00) |
|  | 3 | 0.67 (0.17, 0.88) | 0.76 (0.23, 0.95) | 0.86 (0.29, 0.98) | 0.91 (0.34, 1.00) |
|  | 4 | 0.39 (0.13, 0.88) | 0.52 (0.19, 0.95) | 0.62 (0.24, 0.97) | 0.73 (0.30, 0.99) |
| PSMA-PET/mpMRI | 1 | 0.59 (0.25, 0.95) | 0.68 (0.31, 1.00) | 0.80 (0.35, 1.00) | 0.87 (0.40, 1.00) |
|  | 2 | 0.61 (017, 0.84) | 0.73 (0.24, 0.93) | 0.85 (0.29, 0.98) | 0.90 (0.34, 1.00) |
|  | 3 | 0.77 (0.26, 0.93) | 0.84 (0.32, 0.99) | 0.88 (0.36, 1.00) | 0.94 (0.41, 1.00) |
|  | 4 | 0.48 (0.22, 0.88) | 0.60 (0.28, 0.95) | 0.70 (0.33, 0.98) | 0.80 (0.38, 1.00) |
